# Complete traumatic fracture-dislocation L3-L4 of the lumbar spine

**DOI:** 10.12669/pjms.295.3783

**Published:** 2013

**Authors:** Hassan Reza Mohammadi, Shokrollah Zandi

**Affiliations:** 1 Hassan Reza Mohammadi, Department of Neurosurgery, Imam Hossein Hospital, Shahid Beheshti, University of Medical Sciences, Tehran, Iran.; 2 Shokrollah Zandi, Department of Medicine, Kurdistan University of Medical Sciences, Sanandaj, Iran. Neurosurgeon Resident in Imam Hossein Hospital, Shahid Beheshti, University of Medical Sciences, Tehran, Iran.

**Keywords:** Spinal Fractures, Spinal Cord Injury, Decompression

## Abstract

Complete traumatic fracture-dislocation of the lumbar spine is a rare spinal injury often leading to death. Surgical intervention is an effective treatment modality to decrease mortality. Our patient was a 16 year old boy who had a collision caused by fall from a construction material lifter. He was referred to our hospital with waist trauma. Later, the patient underwent posterior surgical decompression during which vertebral column restoration by long segment fixation was performed. After surgery there were no significant changes in the patient condition.

## INTRODUCTION

There is a growing number of studies indicating the role of 'secondary injury' in spinal cord injuries.^1^ Fracture dislocations of the thoracic and lumbar spine comprise less than 3% of all spinal injuries. They are responsible for unstable lesions with a neurologic deficit and canal compromise. Severe trauma can be responsible for a complete spinal anterior dislocation with a 100% anterior slipping of the vertebral body.^2^ Herein we present a case of this very rare and severe lesion.

## CASE REPORT

A 16 year old boy had a collision caused by fall from a construction material lifter. He was referred to our hospital with waist trauma. At the time of admission the patient was conscious with GCS: 15, BP: 120/80, PR:85, T:37.3 and RR: 16. The patient complained of difficulty in the movement of lower limbs and its relevant decreased sensation. Observation of waist showed minor step-like deformity in the lumbar region. In the initial examination strength in the lower extremity was 0/5 and that of proximal extremity was 3/5. There was lack of sensation, pain, and touch in the lower extremities and the patient had urinary and bowel incontinence. Lateral radiograph and computed tomographic scan of the lumbar spine revealed complete dislocation of the L3-L4 vertebra. L3 had complete dislocation toward anterior part of L4 vertebra and was located in front of it which caused shortening of the vertrbral column. The radiograph showed fracture in the posterior element of the L3 vertebra. At the same time the patient was hemodynamically stable and there was no sign of damage to the interior organs. Later, the patient underwent posterior surgical decompression during which vertebral column restoration by long segment fixation was performed. L3 laminectory and torn dura repair was also undertaken.

 After surgery there was no significant change in the patient’s condition, hence the patient went under restorative therapy which resulted after six months in increasing proximal strength of the lower extremities (4/5) as well as distal strength of the lower extremities (2/5).

.

**Fig.1:**
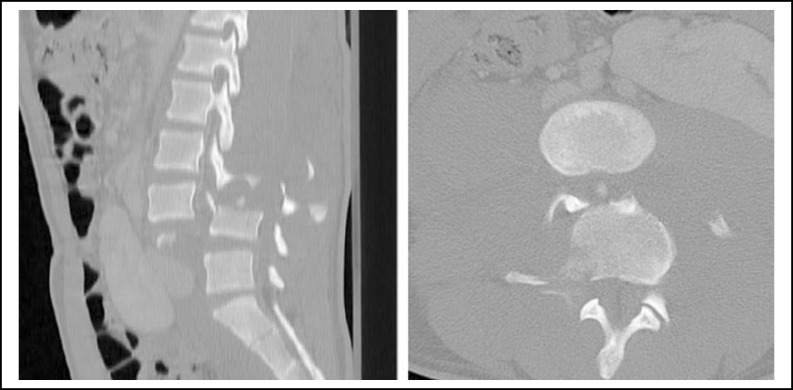
CT scan showing complete dislocation in the L3 – L4 in the axial view and its Sagittal restoration

## DISCUSSION

Complete dislocation and fracture of vertebral column is reported rarely. Fracture dislocation of the thoracic and lumbar vertebral column comprised 3% of the injuries related to the vertebral column. This kind of fractures usually occurs along with instability in the vertebral column, neurologic and spinal column involvement. Severe trauma could lead to complete anterior dislocation and full (100%) vertebral slippage. Complete dislocation of the vertebral column is a rare fracture among fracture dislocations in the vertebral column.^2^

 Severe traumatic spinal fracture dislocations such as that seen in our patient are infrequent because these patients usually die at the scene due to severe trauma and hypovolemic shock resulting from bleeding. Severe axial force after pressure from back to the front caused anterior dislocation. Complete fracture dislocation of the vertebra usually causes complete neurologic defect below the level of spinal injury.^3^

 Fracture dislocation is the most common unstable vertebral injury involving three vertebral columns. The shearing force of which has been classified by Denis which involves translational displacement of vertebra, transection of spinal cord and tear of dura membrane, and almost result in the complete spinal cord injury.^4^

 High quality computerized tomography of the vertebral column is the best assessment tool for stability of the vertebral column. In unstable fracture dislocation of the vertebral column, surgical intervention for decompression of neurological elements and restoration of normal and stable vertebral column is recommended.^5^
